# Sex and gender classification in clinical decision-support tools: a cross-sectional review of tools on MDCalc

**DOI:** 10.1186/s13293-026-00946-1

**Published:** 2026-06-28

**Authors:** Brototo Deb, Timothy Lim, Ian K. Everitt, Carl G. Streed

**Affiliations:** 1 Department of Cardiovascular Medicine, Brigham and Women’s Hospital, Harvard Medical School, Boston, MA USA; 2 Faculty of Pharmacy, University of Toronto, Toronto, Canada; 3 Division of Cardiology, Johns Hopkins University School of Medicine, Baltimore, MD USA; 4 Department of Medicine, Boston University Chobanian and Avedisian School of Medicine, Boston, MA USA; 5 GenderCare Center, Boston Medical Center, 801 Massachusetts Ave. Rm 2082, Boston, MA 02118 USA

**Keywords:** Sex, Gender, Clinical tools, Sex and gender measurement, Health equity

## Abstract

**Background:**

As algorithm-based decision support tools become increasingly integrated into clinical workflows, particularly with the popularization of artificial intelligence, the conceptualization and operationalization of demographic variables such as sex and gender have implications for how these variables are measured and interpreted. Misalignment between variable labels, response categories, and underlying data sources may introduce measurement error or ambiguity in algorithm inputs, potentially affecting clinical interpretation and downstream decision-making. This concern reflects issues of variable specification rather than terminology alone. The objective of this study is to evaluate how sex and gender are labeled, operationalized, and aligned with underlying source data in clinical decision-support tools.

**Methods:**

In May 2025, we conducted a cross-sectional review of clinical tools available on MDCalc, a widely-used online repository of clinical decision-support tools derived from biomedical research and clinical guidelines. We assessed tools with a demographic input labeled as sex, gender, or both. Main measures were the presence and operationalization of sex or gender variables within tool interfaces and corresponding primary references. Variable labeling (sex or gender), response categories (e.g., male/female or man/woman), reported data collection methods in primary references, and concordance between tool interfaces and source literature. Tools were also qualitatively categorized by potential clinical impact (high, medium, low).

**Results:**

Among 821 tools screened, 84 (10.2%) included a sex or gender variable. Of these, 69 (82.1%) labeled the variable as sex and 15 (17.9%) as gender. Binary response categories were used in 83 tools (98.8%). Sex–gender conflation occurred in 16 tools (19.0%), reflecting inconsistencies between variable labels and response categories used as inputs. Primary references were available for 76 tools (90.5%); among those that explicitly reported sex or gender collection, 18 (25.7%) demonstrated sex–gender conflation. Most tools were classified as having medium (51.2%) or high (33.3%) potential clinical impact.

**Conclusion:**

Sex and gender are inconsistently labeled and operationalized in clinical decision-support tools. One in five tools demonstrated misalignment between variable labels, response categories, and underlying source data. Greater conceptual clarity and transparency of sex and gender variables may reduce ambiguity and strengthen the validity, interpretability, and equity of algorithm-based clinical decision tools.

## Highlights

Researchers examined 821 clinical tools on MDCalc, a website used by millions of healthcare professionals globally, and found that nearly 1 in 5 tools asking about sex or gender used these terms incorrectly or interchangeably.

Almost all calculators only offered “male” or “female” choices, excluding people who identify outside these categories, such as transgender or non-binary individuals.

The confusion between sex and gender in these tools could lead to inaccurate results for certain patients, potentially affecting diagnosis, treatment decisions, and medication dosing.

This research is one of the first to systematically examine how sex and gender are handled across a major clinical decision-support platform, revealing a previously unrecognized problem.

The findings suggest medical calculator developers need clearer guidelines to ensure these widely-used tools collect the right information and provide accurate results for all patients.

## Abstract (plain language summary)

Doctors often use online calculators to help make medical decisions, such as estimating your risk for heart disease or determining the right medication dose. Many of these tools ask whether you are “male” or “female.” But researchers have found that these tools often mix up two different things sex and gender. Sex refers to physical characteristics you were born with, like hormones and reproductive organs, as well as your legal status. Gender is about how you identify and express yourself as a man, woman, both, neither, or somewhere in between. For most people, these match up, but for some people, including transgender individuals, they may differ. Researchers looked at 821 medical calculators on a popular website called MDCalc, which millions of doctors use worldwide. They found that about 1 in 10 tools asked about sex or gender, and nearly 1 in 5 of these tools used the terms incorrectly. For example, tools asked about “gender” but only offered “male/female” as choices, and almost all tools only gave two options, with no choices for people who do not fit neatly into these categories. When these tools mix up sex and gender, it can create confusion about which information healthcare providers should enter. This may lead to less accurate results, particularly for transgender or gender-diverse patients. As medical tools become more advanced, ensuring they correctly ask about and use patient information, including sex and gender, is important for providing accurate, fair care to everyone.

## Introduction

Sex and gender are routinely collected demographic variables used for healthcare delivery and biomedical research [1, 2]. A current understanding of sex, informed by gender studies in health-related research, recognizes it to be a multidimensional construct encompassing sex assignment at birth (i.e., the sex someone is assigned at or before birth, typically based on observation of external genitalia that is interpreted through prevailing medical and cultural standards), legal status (i.e., the sex that is recognized under the law and appears on identity documents), and biological characteristics, including chromosomes, hormones, gonads and reproductive anatomy [2–4]. While sex assigned at birth is frequently collected in health-related research, it is a classification made at birth primarily on observed anatomical characteristics, and should not be considered a comprehensive measure of sex.

Gender, by contrast, is a social construct and operates across dimensions including identity, expression and modality [5–7]. Gender identity reflects a person’s internal sense of identity, including being a woman, man, both, neither or another identity somewhere along the gender spectrum [5]. An individual’s external presentation can describe their gender expression, whereas gender modality is the relationship between an individual’s sex assigned at birth and their current gender identity [4–7]. Beyond these individual-level dimensions, gender also operates as a societal structure that organizes social norms, power relations, institutional practices, and access to resources. Therefore, gender influences health not only through individual experiences and identities but also through broader social and institutional processes [7]. 

For many people, their gender modality is cisgender, meaning their gender identity corresponds with their sex assigned at birth. This alignment may contribute to a common assumption that sex and gender are interchangeable, despite representing distinct aspects of the human experience. Consequently, sex and gender are frequently conflated [2, 4]. However, for some sexual and gender minority (SGM) individuals, these identities do not align [8, 9]. When sex and gender are used interchangeably in clinical data systems or research datasets, the resulting ambiguity may compromise the reliability and interpretability of demographic variables used in clinical analyses and decision tools [10, 11]. Moreover, when different aspects of sex and/or gender of relevance are not well defined, that can contribute, yet again, to construct conflation. For instance, data on gender identity may be used to infer aspects of gender expression, even though these represent separate aspects of gender, and can undermine the validity and precision of analyses [2, 4]. 

These concerns are particularly salient for clinical decision-support tools. Many clinical algorithms incorporate demographic variables, such as sex, as structured inputs that influence diagnostic or prognostic estimates. Clinical calculators are algorithmic decision-support tools that translate patient-specific inputs, such as demographic characteristics, lab values, vital signs, comorbidities, and symptoms, into estimates of diagnosis, disease severity, prognosis, or treatment recommendations [12]. For example, the CHA2DS2-VASc score incorporates sex as a variable that influences stroke risk estimates in individuals with atrial fibrillation [13]. If the sex variable is interpreted or recorded inconsistently with how it was defined in derivation cohorts, the resulting input may not accurately reflect the intended parameter. Such discrepancies may lead to misclassification of patients, particularly transgender or gender non-binary patients, and potentially influence clinical recommendations, including anticoagulation decisions. This example illustrates how ambiguity in the specification of sex and gender variables can affect the interpretation and reliability of clinical decision-support algorithms rather than representing a purely semantic distinction.

With the increasing integration of artificial intelligence (AI) and machine learning into clinical care, algorithm-based tools are likely to become more prominent in diagnostic and treatment workflows [14]. As predictive models and decision-support systems incorporate demographic variables as structured inputs, careful attention to how these variables are defined, labeled, and operationalized becomes increasingly important. Inconsistent specification of sex or gender variables across datasets, model development studies, and clinical interfaces can introduce ambiguity and complicate the interpretation of model outputs. These issues may have downstream implications for clinical care, including risk stratification, treatment recommendations, and the distribution of health care services [15, 16]. 

MDCalc is a widely used digital repository of clinical calculators and tools derived from published biomedical research and clinical guidelines [17]. The platform is consistently rated as one of the most useful medical platforms for healthcare professionals and is used by millions of clinicians worldwide, with more than 80% of users in the United States [17]. The clinical calculators and tools, hereon referred to as tools, compiled on MDCalc may improve decision-making and enhance healthcare delivery [12]. Many of these tools include demographic variables, such as sex or gender, as inputs that influence calculated scores or predicted outcomes. In these contexts, alignment between the variables used in the clinical interface and those defined in the underlying source literature is important for the accurate application and interpretation of the algorithm. Despite the widespread use of these tools, the extent to which sex and gender variables are consistently specified between tool interfaces and their primary references has not been systematically evaluated.

Accordingly, this study aimed to identify clinical tools on MDCalc that include sex or gender as input variables, characterize how these variables are labelled and categorized, assess concordance between tool interfaces and the underlying primary literature, and classify identified tools according to their potential clinical impact.

## Materials and methods

### Study design

This was a cross-sectional study that evaluated clinical decision-support tools made publicly available on the host platform, MDCalc. Data were extracted in May 2025. Tools were eligible for inclusion if they were available in English on MDCalc and included a demographic input explicitly labeled as “sex” or “gender” or used categories with “male/female” or “man/woman” in their calculation schema. Tools without sex or gender-related inputs were excluded.

### Data collection and variable definitions

For each eligible tool, data were extracted from the tool interface and its corresponding primary reference when available. Extracted variables from the tool included the demographic label and the response categories presented (e.g., male/female, man/woman).

From the primary reference of the tool, the terminology used to describe the demographic variable, the response categories, and the reported method of data collection were collected. Two reviewers independently abstracted the data using a structured data collection form. Discrepancies were resolved through discussion and consensus.

Sex–gender conflation was defined a priori as any instance in which the label of a demographic variable did not align with its operationalization, including labeling gender while using sex categories (i.e., male/female) or labeling sex while using gender identity categories (i.e., man/woman).

### Impact classification

Each tool was qualitatively categorized as having low, medium, or high potential clinical impact based on predefined criteria. This impact classification was intended to contextualize the implications of variable misclassification and was not intended to measure clinical effectiveness of the tool or serve as a definitive ranking. The constructs that defined the criteria included clinical significance and use in practice, national and/or international recognition, scope of applicability (broad vs. niche use), impact on clinical outcomes (e.g., morbidity vs. mortality), and context of use (e.g., routine care versus time-limited contexts like the COVID-19 pandemic). COVID-specific tools were categorized as low impact due to the transition of COVID-19 from a federal public health emergency during the initial pandemic phase into an endemic infection.

### Data analysis

Descriptive statistics were used to summarize sex and gender labeling and categorization across the tool interfaces and their corresponding reference sources. Analyses looked at the proportion of tools that used sex and gender labels, frequency of different categories (e.g., male/female vs. man/woman), association between data source (self-report versus chart review) and categories used, and the distribution of category choice and impact tier. Visualization of these flows is portrayed through a multi-level flowchart and a vertical Sankey diagram. All data were managed in Microsoft Excel. Diagrams were created using Python’s Graphviz and Plotly libraries in a Jupyter environment.

## Results

### Study sample

A total of 821 clinical decision-support tools available on MDCalc were screened for eligibility in May 2025. Of these, 84 (10.2%) met inclusion criteria. Among the 84 included tools, 69 (82.1%) included a demographic input labeled as sex, and 15 (17.9%) included a gender input. Across the MDCalc platform, a total of 83 tools (98.8%) used binary response categories of “male/female,” while 1 tool (1.2%) used man/woman. Overall, 16 clinical tools (19.0%) met criteria for sex-gender conflation. Specifically, 15 tools used “male/female” categories when asking for gender and 1 tool used man/woman categories for sex (Table 1). No tools were identified with more than binary categories.

**Table 1:** Scores conflating sex and gender on MDCalc website (*n* = 16)

Link to the score	Description
**Calculator with categories of gender encoded as “male/female”**	
https://www.mdcalc.com/calc/10030/choles-score-duration-laparoscopic-cholecystectomy	The CholeS Score for Duration of Laparoscopic Cholecystectomy predicts operative time of laparoscopic cholecystectomy
https://www.mdcalc.com/calc/10046/denver-hiv-risk-score	The Denver HIV Risk Score predicts probability of undiagnosed HIV infection
https://www.mdcalc.com/calc/10094/galad-model-hepatocellular-carcinoma-hcc	The GALAD Model for Hepatocellular Carcinoma (HCC) diagnoses HCC based on serum biomarkers
https://www.mdcalc.com/calc/10104/apfel-score-postoperative-nausea-vomiting	The Apfel Score for Postoperative Nausea and Vomiting predicts risk of postoperative nausea and vomiting (PONV)
https://www.mdcalc.com/calc/10465/improve-bleeding-risk-score	The IMPROVE Bleeding Risk Score evaluates bleeding risk at hospital admission
https://www.mdcalc.com/calc/10471/acute-renal-failure-cardiac-surgery-thakar-score	The Acute Renal Failure after Cardiac Surgery (Thakar Score) predicts risk of acute renal failure (ARF) requiring dialysis after cardiac surgery
https://www.mdcalc.com/calc/10478/vesicoureteral-reflux-index-vurx	The Vesicoureteral Reflux Index (VURx) predicts resolution of VUR in children
https://www.mdcalc.com/calc/2157/gap-index-idiopathic-pulmonary-fibrosis-ipf-mortality	The GAP Index for Idiopathic Pulmonary Fibrosis (IPF) Mortality provides 1, 2, and 3-year mortality estimates for IPF patients
https://www.mdcalc.com/calc/3803/maggic-risk-calculator-heart-failure	The MAGGIC Risk Calculator for Heart Failure estimates 1- and 3- year mortality in patients with heart failure
https://www.mdcalc.com/calc/3808/seattle-heart-failure-model	The Seattle Heart Failure Model predicts survival in heart failure
https://www.mdcalc.com/calc/3928/endotracheal-tube-ett-depth-tidal-volume-calculator	The Endotracheal Tube (ETT) Depth and Tidal Volume Calculator estimates depth of optimal ETT placement and target tidal volume by height
https://www.mdcalc.com/calc/3992/stop-bang-score-obstructive-sleep-apnea	The STOP-BANG Score for Obstructive Sleep Apnea screens for obstructive sleep apnea
https://www.mdcalc.com/calc/4000/findrisc-finnish-diabetes-risk-score	The Finnish Diabetes Risk Score identifies patients at high risk for type 2 diabetes
https://www.mdcalc.com/calc/4001/cambridge-diabetes-risk-score	The Cambridge Diabetes Risk Score predicts risk of having previously undiagnosed type 2 diabetes
https://www.mdcalc.com/calc/4020/american-diabetes-association-ada-risk-calculator	The American Diabetes Association (ADA) Diabetes Risk Calculator predicts risk of undiagnosed diabetes to determine who should be screened
**Calculator with categories of sex encoded as man/woman**	
https://www.mdcalc.com/calc/10544/canadian-association-radiologists-osteoporosis-canada-caroc-system	The Canadian Association of Radiologists and Osteoporosis Canada (CAROC) System assesses 10-year absolute fracture risk

### Primary references

Primary references were available for 76 of the 84 tools (90.5%). Among these, 4 references (5.3%) did not explicitly specify sex or gender, and 2 references (2.6%) used the terms interchangeably. Additionally, among the 70 tools with primary references that explicitly mentioned sex or gender, 18 (25.7%) had sex-gender conflation (Table 2).

**Table 2:** Tools conflating sex and gender in their primary reference (*n* = 18)

MD calc link	Description of the score
**Calculator with categories of gender encoded as male/female**	
https://www.mdcalc.com/calc/10030/choles-score-duration-laparoscopic-cholecystectomy	The CholeS Score for Duration of Laparoscopic Cholecystectomy predicts operative time of laparoscopic cholecystectomy
https://www.mdcalc.com/calc/10046/denver-hiv-risk-score	The Denver HIV Risk Score predicts probability of undiagnosed HIV infection
https://www.mdcalc.com/calc/10104/apfel-score-postoperative-nausea-vomiting	The Apfel Score for Postoperative Nausea and Vomiting predicts risk of postoperative nausea and vomiting (PONV)
https://www.mdcalc.com/calc/10227/orbit-bleeding-risk-score-atrial-fibrillation	The ORBIT Bleeding Risk Score for Atrial Fibrillation predicts bleeding risk in patients on anticoagulation for afib, similar to HAS-BLED
https://www.mdcalc.com/calc/10471/acute-renal-failure-cardiac-surgery-thakar-score	The Acute Renal Failure after Cardiac Surgery (Thakar Score) predicts risk of acute renal failure (ARF) requiring dialysis after cardiac surgery
https://www.mdcalc.com/calc/10478/vesicoureteral-reflux-index-vurx	The Vesicoureteral Reflux Index (VURx) predicts resolution of VUR in children
https://www.mdcalc.com/calc/10504/garfield-af	GARFIELD-AF predicts mortality, stroke, and bleeding in patients w/ and w/out anticoagulation
https://www.mdcalc.com/calc/1236/tash-score-trauma-associated-severe-hemorrhage	The Trauma Associated Severe Hemorrhage (TASH) Score is a derived (and then validated) score from a database of over 35,000 German trauma patients to predict the need for massive transfusion
https://www.mdcalc.com/calc/1757/opioid-risk-tool-ort-narcotic-abuse	The Opioid Risk Tool for Narcotic abuse estimates risk of opioid-related aberrant behaviors based on patient history
https://www.mdcalc.com/calc/3166/ripasa-score-acute-appendicitis	The RIPASA Score for Acute Appendicitis diagnoses appendicitis with lab values and clinical findings, specifically for Asian populations
https://www.mdcalc.com/calc/3803/maggic-risk-calculator-heart-failure	The MAGGIC Risk Calculator for Heart Failure estimates 1- and 3- year mortality in patients with heart failure
https://www.mdcalc.com/calc/3808/seattle-heart-failure-model	The Seattle Heart Failure Model predicts survival in heart failure
https://www.mdcalc.com/calc/3992/stop-bang-score-obstructive-sleep-apnea	The STOP-BANG Score for Obstructive Sleep Apnea screens for obstructive sleep apnea
https://www.mdcalc.com/calc/4001/cambridge-diabetes-risk-score	The Cambridge Diabetes Risk Score predicts risk of having previously undiagnosed type 2 diabetes
https://www.mdcalc.com/calc/801/cha2ds2-vasc-score-atrial-fibrillation-stroke-risk	The CHA2DS2-VASc Score for for Atrial Fibrillation Stroke Risk calculates stroke risk for patients with atrial fibrillation, possibly better than the CHADS2 score
**Calculator with categories of sex encoded as man/woman**	
https://www.mdcalc.com/calc/10371/covid-19-inpatient-risk-calculator-circ	The COVID-19 Inpatient Risk Calculator (CIRC) predicts likelihood of inpatient mortality or severe disease progression in COVID patients
https://www.mdcalc.com/calc/10472/relative-fat-mass-rfm	The Relative Fat Mass (RFM) estimates whole body fat percentage among adults
https://www.mdcalc.com/calc/38/framingham-risk-score-hard-coronary-heart-disease	The Framingham Coronary Heart Disease Risk Score estimates risk of heart attack in 10 years

### Data sources for sex and gender variables

**Fig. 1:**
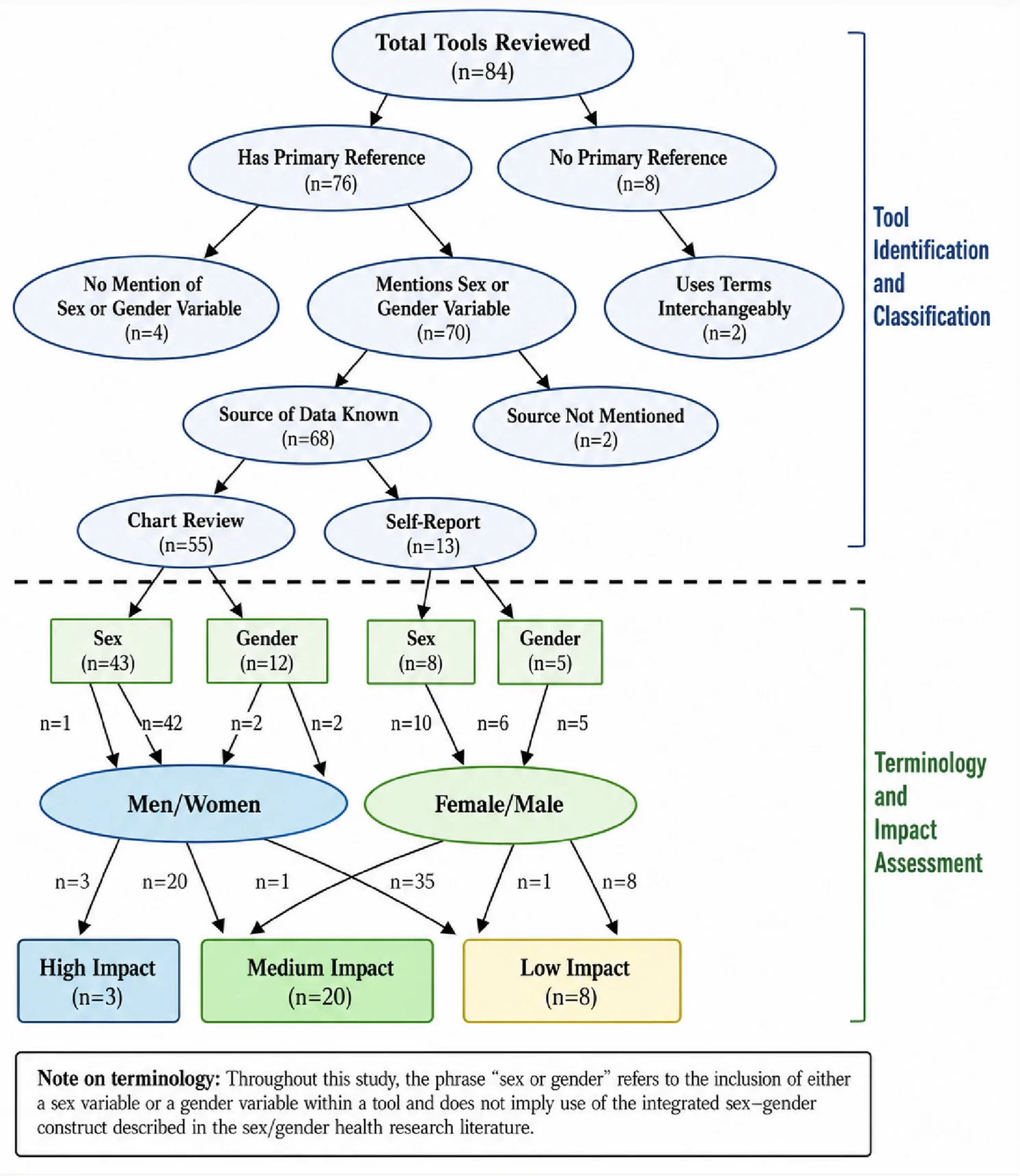
Classification of cardiovascular risk prediction tools by inclusion of sex or gender variables, terminology used, and impact category. Flow diagram illustrating identification of tools, determination of data source (chart review or self-report), classification of variables as sex or gender, terminology used in the tool (female/male vs. men/women), and resulting impact categorization. Throughout this study, “sex or gender” denotes the inclusion of either a sex variable or a gender variable and does not refer to the integrated sex–gender construct

Among tools with primary references available for review, the mode of data collection was also examined (Fig. 1). Of the 70 tools with primary references that explicitly reported sex or gender, 55 references (78.6%) collected sex or gender data by abstraction through electronic health record(s) (EHR). 13 references (18.6%) collected data through self-reporting, and 2 references (2.9%) did not specify the mode of data collection. Among the 13 tools utilizing self-reported gender data, none of the references used man/woman as categories. Among the 55 tools utilizing EHR review, 12 tools (21.8%) collected gender data, and only two (3.6%) used the categories of man/woman.

**Fig. 2:**
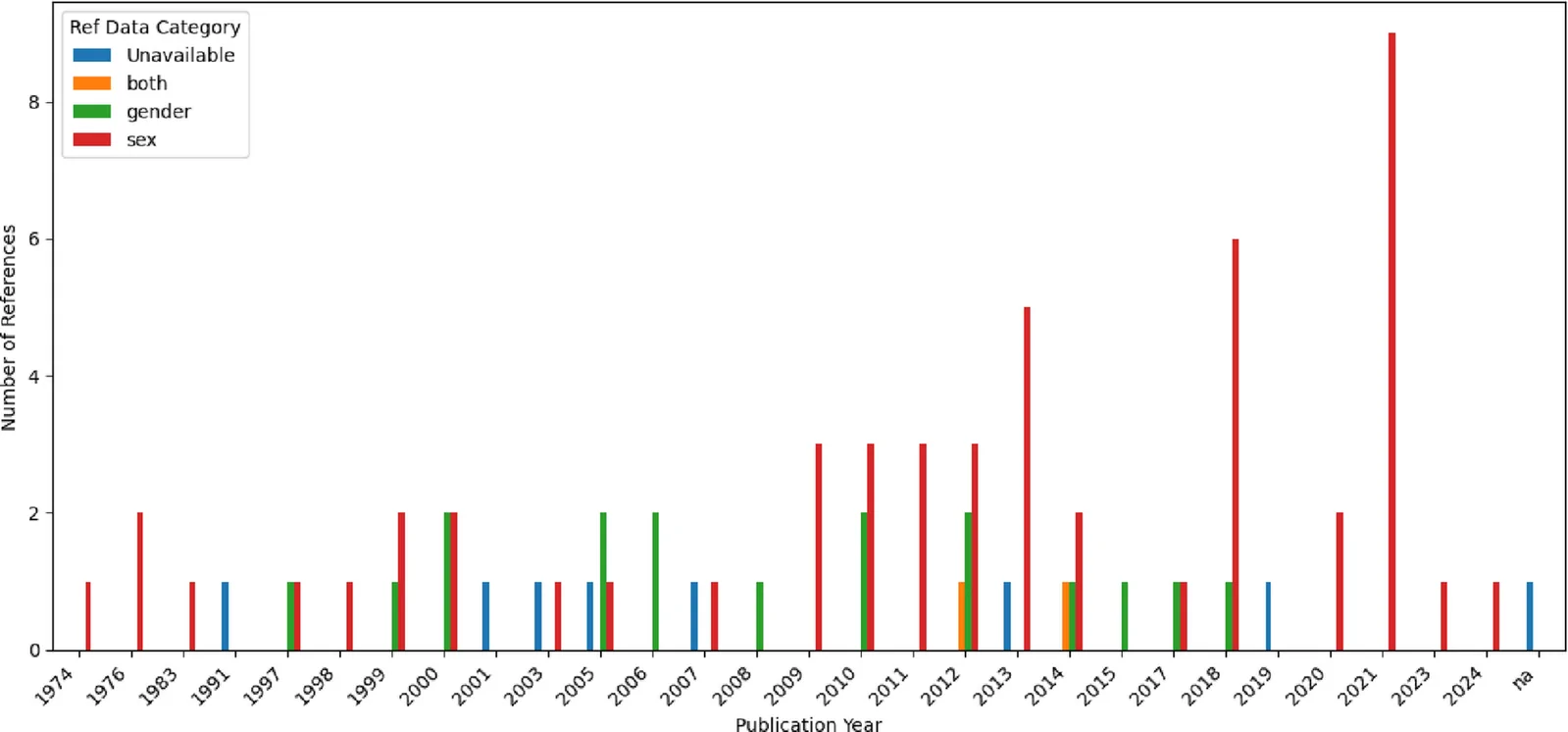
Number of references using sex and gender categories by publication year

Figure 2 shows the distribution of primary references used to develop the included clinical tools according to whether the demographic variable was described as sex or gender, stratified by year of publication. Across publication years, most references described the variable using sex-based terminology, and few used gender-based terminology. This pattern suggests that the literature underlying many clinical decision-support tools continues to conceptualize these demographic variables primarily through sex assigned at birth. However, the presence of some references using gender indicates variability in how these constructs are defined and reported in the studies informing these tools.

**Fig. 3:**
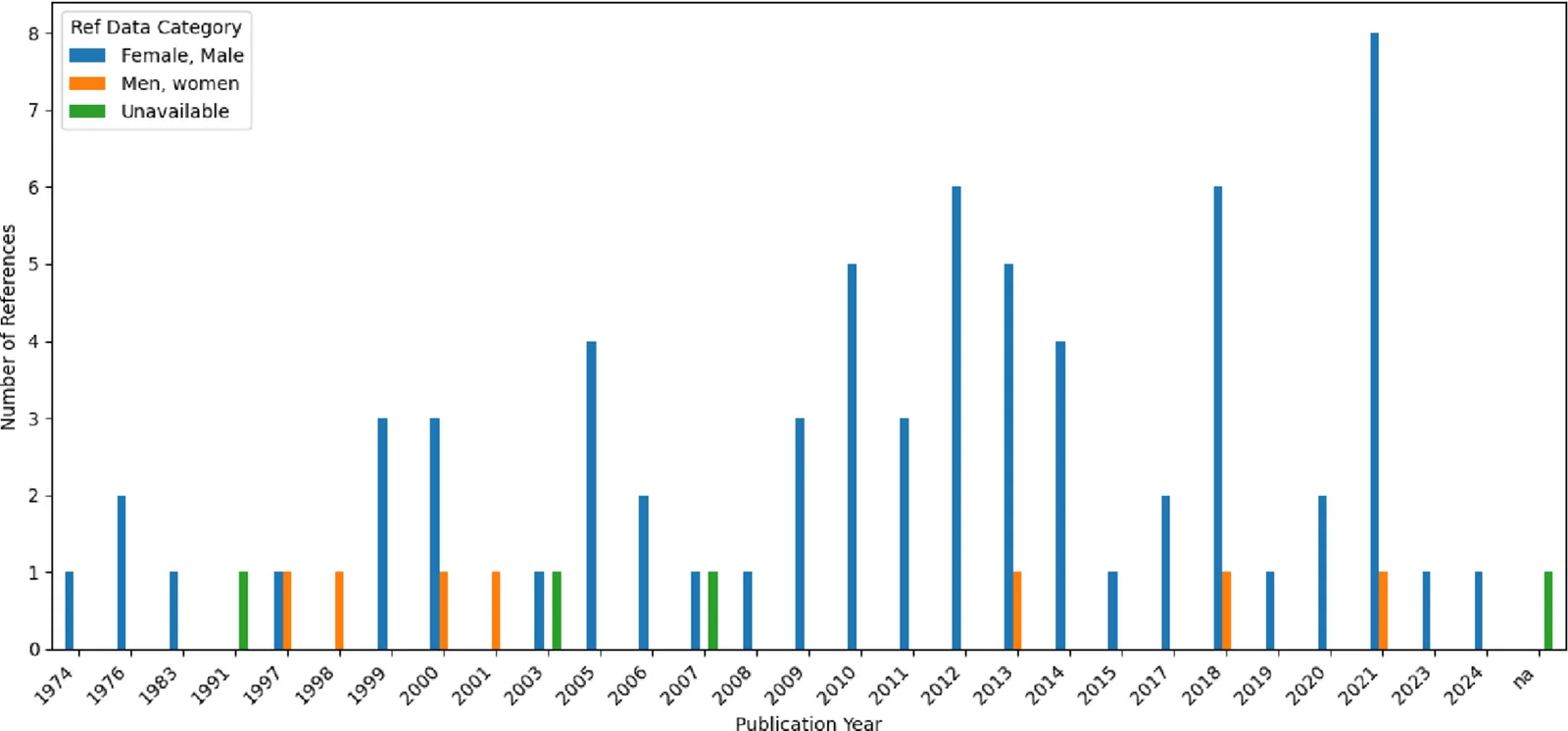
Number of references using male/female or man/woman categories by publication year

Figure 3 illustrates the response categories used in primary references by publication year. Across all years, most studies operationalized male/female categories. Only a small number of references used man/woman categories, which align more closely with gender identity terminology. Even when gender-based terminology was used in study descriptions, the operational response categories frequently remained male/female, highlighting inconsistencies between conceptual labels and underlying measurement.

### Impact categorization

Most of the clinical tools were classified as medium impact (*n* = 43, 51.2%), followed by high impact (*n* = 28, 33.3%) and low impact (*n* = 13, 15.5%). Of the 5 tools that categorized data as man/woman in their primary literature, 4 were high or medium impact. Tools that operationalized demographic variables using “male/female” categories were also predominantly classified as high or medium impact (Fig. 1).

## Discussion

In this cross-sectional analysis of clinical decision-support tools on MDCalc, sex and gender variables were often inconsistently labeled or operationalized across tool interfaces and their corresponding primary references. Approximately one-fifth of tools demonstrated misalignment between the variable label and response categories at the interface level, and more than one-quarter used sex or gender inputs interchangeably across the primary sources. These patterns were observed across tools with varying levels of potential clinical impact. Together, these findings suggest that demographic variables used as algorithm inputs are not always specified consistently between derivation studies and clinical tool interfaces, introducing potential ambiguity in how inputs should be interpreted when applying clinical decision-support algorithms in practice.

Despite increasing awareness of the distinction between sex and gender in biomedical research, inconsistent reporting and operationalization of these variables remain common across the research ecosystem. Progress toward systematically integrating sex and gender considerations into health research has been uneven: clinical research more often includes sex, population health research often examines gender, and biomedical research has historically incorporated either inconsistently [18]. Although inclusion of sex and gender in study design has risen, the number of studies rigorously examining sex- or gender-specific effects remains limited [19]. For example, a 2016 review of randomized controlled trials in high-impact international journals found that only 6% of studies including both men and women examined interactions between sex or gender and other variables of interest [20]. These broader patterns in research design may contribute to downstream inconsistencies when study-derived variables are translated into clinical algorithms and decision-support tools. In our study, the use of sex rather than gender categorization became more common in recently developed tools, which may reflect increasing recognition that sex and gender represent distinct constructs and efforts to align algorithm inputs more closely with presumed biological mechanisms, such as genetic, hormonal, or physiologic differences captured within derivation cohorts. However, sex assigned at birth alone may not fully capture dynamic factors over the life course or gender-related social and structural factors that also influence health outcomes.

There is broad consensus among medical professional organizations that collecting sexual orientation and gender identity (SOGI) data is important for identifying health disparities, improving patient-centered care, and supporting inclusive biomedical research [21–24]. Electronic health record (EHR) platforms provide an opportunity to capture self-reported gender identity, enabling researchers and clinicians to examine variation in health outcomes and care delivery across populations. Recognizing the importance of this data, federally funded health centers have been required since 2016 to report SOGI data through the Uniform Data System, and in 2018 the Centers for Medicare and Medicaid Services required certified EHR systems to include the capability to record SOGI data as part of the Meaningful Use criteria [25]. Despite these advancements, challenges remain, including variability in how SOGI data are collected within EHR systems, inconsistent staff training, and limited standardization of clinical workflows [26, 27]. Because many clinical decision-support tools are developed using large observational datasets derived from EHRs or population-based cohorts, the specification of demographic variables in these data sources directly influence the reliability of downstream clinical algorithms. Ensuring that sex, gender identity, and related demographic variables are clearly defined and consistently captured is essential for developing clinical tools that are both clinically valid and applicable across diverse patient populations.

Our findings also highlight the widespread reliance on binary categorization within clinical support tools. All tools evaluated in this study employed binary response categories (e.g., male/female or man/woman), and in several instances, gender-based variables were operationalized using sex-based categories. These issues are particularly relevant for transgender and gender-diverse individuals, for whom discordance between gender identity and sex assigned at birth may complicate the interpretation of algorithm inputs derived from historical cohorts. However, incorporating categories beyond binary classifications requires several considerations. Expanding response options without adequate representation in derivation cohorts or validation studies may not improve predictive accuracy and could introduce additional uncertainty. Future development of more inclusive algorithms will depend on standardized SOGI data collection, adequate representation of gender-diverse populations, and empirical evaluation of whether additional demographic categories improve model performance or clinical utility. Although future algorithms may benefit from incorporating more specific dimensions of sex and gender, such as hormonal exposure, anatomy, or gender-related social experiences, these variables must be routinely captured, standardized, and shown to improve prediction beyond existing models.

The mismatch between variable labels and response categories observed in this study also reflects a broader issue of construct validity in algorithm design. Labeling an input field as gender while limiting options to male or female represents a misalignment between the conceptual variable and the measurement used to capture it. This type of measurement inconsistency can lead to misclassification and may affect the accuracy of risk stratification. Our findings align with prior work demonstrating that dichotomizing or conflating sex and gender variables can introduce confounding and misclassification in statistical inferences [26]. Ensuring that variable labels, response options, and underlying definitions are aligned is therefore an important aspect of maintaining the validity and reliability of clinical decision-support tools.

Our findings also raise broader questions about the role of demographic variables in clinical algorithms. For example, the 2024 European Society of Cardiology guidelines for atrial fibrillation management revised the CHA2DS2-VASc score by removing sex as an independent risk factor, recognizing that female sex functions primarily as a modifier of other stroke risk factors rather than as an independent predictor [28–30]. This evolution illustrates how the role of demographic variables in risk prediction models may change as evidence accumulates and highlights the importance of continuously evaluating how such variables are incorporated into clinical algorithms. Similar discussions have occurred regarding the use of race in clinical prediction tools, where race is increasingly recognized as a proxy for structural and social determinants of health rather than an intrinsic biological determinant [31–34]. These examples underscore the importance of carefully specifying demographic variables used as algorithm inputs and evaluating whether their inclusion improves predictive validity and clinical utility.

An important future direction is determining whether clinical algorithms can move beyond static demographic categories toward more direct measurement of the biological, clinical, and social processes these variables represent. For example, recent cardiovascular risk prediction efforts have incorporated neighborhood-level socioeconomic indices to better capture contextual factors influencing health outcomes. Similar approaches may allow future tools to represent relevant dimensions of sex and gender more precisely while preserving clinical usability. Although sex and gender are interconnected, and future models may incorporate interactions among biological, behavioral, and social dimensions when predictive, increasing model complexity should be guided by evidence that additional variables improve clinical decision-making.

As algorithm-based decision tools and AI systems become more widely integrated into clinical workflows, greater attention to the specification and transparency of input variables will be necessary to ensure reliability and interpretability. Clarifying how demographic variables such as sex and gender are defined, measured, and implemented within algorithms is therefore not a semantic exercise but a fundamental issue of data integrity and model validity. Improving consistency and transparency across datasets, research studies, and clinical interfaces may strengthen the performance and equitable application of algorithm-based decision tools.

This study has several important strengths. To our knowledge, this represents one of the first systematic evaluations of how sex and gender variables are defined, operationalized, and translated across a major clinical decision-support platform. By evaluating both tool interfaces and their corresponding primary references, we were able to examine not only how demographic variables are reported in clinical research but also how these variables are implemented at the point of care. Given the increasing integration of algorithm-based decision tools into clinical workflows, these findings provide timely insight into opportunities to improve transparency, consistency, and measurement validity in clinical decision-support systems.

### Limitations

This study has several limitations. First, the analysis was descriptive and cross-sectional. We did not evaluate whether sex or gender misclassification alters tool outputs, influences clinical decision-making, or affects patient outcomes. Consequently, these findings should not be interpreted as evidence that the observed inconsistencies directly affect care quality or health outcomes. Second, terminology and reporting standards for SOGI data have evolved over time. Some of the primary references underlying the tools evaluated in this analysis predate contemporary guidance on demographic data collection and reporting. As a result, some observed inconsistencies may reflect historical research practices rather than current standards. Third, the analysis was restricted to clinical decision-support tools available on the MDCalc platform. Although this platform is widely used by clinicians, the findings may not be generalizable to tools hosted on other digital platforms, integrated into EHR systems, or implemented in proprietary clinical decision-support software.

## Conclusion

In this cross-sectional analysis of clinical decision-support tools, sex and gender variables were frequently labeled or operationalized inconsistently across one in five tool interfaces and their corresponding primary references. These inconsistencies can introduce ambiguity in how demographic variables are interpreted when used as inputs in clinical algorithms. Addressing these issues is not primarily a matter of terminology but of measurement specification and data quality within clinical algorithms. Improving the consistency and transparency of demographic variable definitions across datasets, research studies, and clinical interfaces may strengthen the reliability and equitable application of algorithm-based decision tools.

## Data Availability

All data are publicly available at https://www.mdcalc.com/. The datasets generated and analysed during the current study are available from the corresponding author on reasonable request.
